# Fibromyalgia: epidemiology and risk factors, a population-based case-control study in Damascus, Syria

**DOI:** 10.1186/s41927-022-00294-8

**Published:** 2022-10-31

**Authors:** Mhd Amin Alzabibi, Mosa Shibani, Tamim Alsuliman, Hlma Ismail, Suja alasaad, André Torbey, Abdallah Altorkmani, Bisher Sawaf, Rita Ayoub, Naram khalayli, Mayssoun Kudsi

**Affiliations:** 1 Faculty of Medicine, Syrian Private University, Mazzeh Street, P.O. Box 36822, Damascus, Syrian Arab Republic; 2 Hematology and Cell Therapy Department, Saint-Antoine Hospital, AP-HP Sorbonne University, Paris, France; 3 Department of Internal Medicine, Hamad Medical Corporation, Doha, Qatar; 4 Department of Surgery, American University of Beirut Medical Center (AUBMC), Beirut, Lebanon; 5 Faculty of Medicine, Damascus University, Damascus, Syria

**Keywords:** Fibromyalgia, Epidemiology, Prevalence

## Abstract

**Background:**

Fibromyalgia is a chronic disease with a high burden. We aim to be the first to investigate the prevalence of fibromyalgia (FM) in Syria and assess its risk factors.

**Methods:**

A self-reported questionnaire was distributed to the public to identify fibromyalgia patients using the American College of Rheumatology (ACR) 2010 modified criteria. Identified cases were matched using age with controls free from rheumatic disorders that were randomly sampled from the same population.

**Results:**

Out of 2966 participants, 350 (11.8%) satisfied the diagnostic criteria. Of these, only 29 (8.2%) were previously diagnosed by a physician, 239 (68.3%) were females, and 69 (19.71%) were diagnosed with depression. Female sex (OR = 1.31), diagnosis of major depressive disorder (OR = 2.62), irritable bowel syndrome (OR = 1.8), and Restless legs syndrome (OR = 1.72) were associated with a higher likelihood of fibromyalgia.

**Conclusion:**

Our study revealed one of the highest prevalence rates of fibromyalgia ever reported in the general population. Efforts must be intensified to increase awareness about this disease in Syrian society as well as among healthcare providers.

**Supplementary Information:**

The online version contains supplementary material available at 10.1186/s41927-022-00294-8.

## Background

Fibromyalgia (FM) is a complex disease characterized by chronic (longer than three months) widespread pain combined with tenderness in muscles and joints all over the body [1]. Non-pain-related symptoms of FM include fatigue that can be exaggerated with minor activities, though inactivity for a long period also increases the symptoms [2], paresthesia with generalized burning and tingling, sleeping disorders with waking up unrefreshed, mood problems, and cognitive disturbances often referred to as “fibro fog” that can interfere with the ability to concentrate, keep attention, and multitasking jobs [1, 2]. The tissue inflammation observed in the patients is a poor explanation for the symptoms, and there is no risk of tissue damage or deformity [3]. The etiology of FM remains unclear, but many physical and/or emotional stressors have been described as triggers for this condition, including emotional and physical trauma, and infections [4]. Many risk factors have been identified, such as genetic predisposition, family history, female sex, and the presence of other painful conditions [2]. FM can be present with other psychiatric comorbidities such as anxiety and/or depression with prevalence rates of up to 30 to 50 percent at the time of diagnosis [5, 6]. Finally, FM is associated with gastrointestinal morbidities such as irritable bowel syndrome (IBS) and gastroesophageal reflux disease (GERD) [7, 8].

FM is considered a common disease to encounter in the clinic with a variation in prevalence in the general population in different countries. In Japan, the prevalence of FM was 2.1%, while in Canada, it was estimated at 3.3% (4.9% in women versus 1.6% in men) [9, 10]. Branco et al. reported the prevalence of FM in 5 European countries and placed it at 4.7% [11]. Another study in Germany estimated the prevalence at 3.8%, with similar rates in men and women, while in Lebanon, Syria's neighboring country, the prevalence of FM was found to be 7% [12, 13]. These differing estimations may reflect differences in study populations, study designs, and measurements. As a result, prevalence estimations can vary, up to 4-folds, depending on the diagnostic criteria applied, which at times exceeded 15% in selected samples [14, 15].

The lack of a gold standard and objective markers to diagnose FM led to the proposal of many diagnostic criteria that rely on clinical assessment and patient reports. In 1990, Wolfe et al. set the first American College of Rheumatology (ACR) criteria for FM diagnosis [16]. It requires a clinician to evaluate 18 body bilateral points and considered the diagnosis of FM to be positive when 11 or more points were positive for pain and tenderness. However, these criteria did not factor in non-pain-related symptoms. Later, the 2010 (ACR) criteria was developed, and then, modified to become self-administered [1]. By 2015, the ACTTION-APS Pain Taxonomy (AAPT) criteria was made, but a new study showed that the ACR 2010 modified criteria offered the best concordance with the clinical judgment in comparison to AAPT criteria [17, 18]. The diagnosis of FM could be complex and lengthy, and there is currently no standard treatment algorithm. Together, these make FM a high burden disease with high-cost health care due to increased usage of pain relief medication and outpatient visits per year in both the pre-and post-diagnosis periods [19]. Hence, FM prevalence evaluation has both clinical and economic importance.

The war in Syria, which has lasted more than a decade, has had a profound impact on the population and has created many economic, social, and educational challenges. Damage to health care infrastructure and loss of medical staff have had a major impact on Syria's health care system. As a result, many have been injured, and approximately 511,000 have died [20]. More than 5.6 million have fled the country [21], and nearly 6.2 million have been internally displaced [22], making this event the second-largest immigration in human history after World War II.

As warfare is considered one of the most psychologically stressful events that anyone can experience [23], with catastrophic effects on the population in both long-term and short-term manners, we aim to be the first study, to our knowledge, to determine FM prevalence in a sample of the Syrian population and find its risk factors, in order to help guide the health care interventions.

## Patients, and methods

### Study design, setting, and participants

A two-stage population-based study was conducted, to determine the prevalence and the associated risk factors of fibromyalgia in the general population of Damascus, Syria.

#### Stage one

Using convenience and snowball sampling, a representative sample of Damascus habitants was questioned by a structured self-administered survey from April 1 to May 1, 2021.

We distributed an electronic and paper-based survey in parallel. The electronic open survey was distributed as a Google Form® using social media platforms (Facebook®, Whatsapp®, and Twitter®) on the authors' own accounts and many groups that concern health, youth, and residents of Damascus. No paid ads were used, and no incentives were offered to the participants.

The paper-based survey was distributed by medical students who handed out the survey face-to-face to the patients, their companions, and workers in the outpatient clinics of Damascus Hospital and Ibn Al Nafees Hospital; two major public hospitals in Damascus, Syria. And then received it on site after the participants completed it.

#### Inclusion criteria

We included the participants who were surveyed from April 1 to May 1, 2021, and met the following conditions: (1) 18 years old or older, (2) living in Damascus at least for the last five years (Additional file 1).

#### Measures

Our survey was composed of two sections:

1. *Socio-demographic characteristics* This section covers nine questions about age, gender, marital status, residence (rural or urban), financial status, employment status, educational level, health insurance, currently diagnosed by a physician with (major depressive disorder, generalized anxiety disorder, Irritable bowel syndrome, Rheumatoid arthritis, Systemic lupus erythematosus, and any other rheumatic disease).
2. *Fibromyalgia questionnaire based on ACR 2010 modified criteria for fibromyalgia diagnosis*[26] This section included questions about Symptom Severity Score (SSS), and widespread pain index (WPI). For SSS, the questions concerned fatigue, waking unrefreshed, and cognitive symptoms (experiencing difficulties thinking or remembering). For each of these symptoms, the participants were asked to rate the level of severity over the past week using the following scale: 0 = No problem; 1 = Slight or mild problems; generally mild or intermittent; 2 = Moderate; considerable problems; often present and/or at a moderate level; 3 = Severe: pervasive, continuous, life-disturbing problems. The survey also included yes and no questions about other symptoms that have occurred within the previous six months such as, headache, lower abdominal pain or cramps, and depression.
3. For WPI, the questions centered on 19 areas in which the participant may have experienced pain over the last week. There was also a question on whether the participant had a disorder that would otherwise explain the pain. Finally, we included a question about the persistence of the aforementioned symptoms for the last three months

#### Stage two

A case-control analysis was designed, as cases were considered the participants who satisfied the modified ACR 2010 fibromyalgia diagnostic criteria (number of cases = 350). The participants who did not meet the criteria of fibromyalgia diagnosis and reported no current diagnosis of rheumatic diseases were considered as controls. In order to optimize the power of the study in detecting any significant associations, four controls per case were selected (number of controls = 1400) randomly from our population sample using frequency matching by categorical age groups (Additional file 2).

#### Statistical analysis

The sample size was calculated using Cochran’s formula. No reference was found that estimated the age of Damascus residents over the age of 18. So, we used the total population number (2,079,000 people) in Damascus city to reduce the error margin [24]. We also used a confidence level of 95%, a margin of error of 1%, and a proportion of 7%, as reported previously in a neighboring population [25]. This resulted in a sample size of 2498.

We analyzed the data using Statistical Package for Social Sciences version 25.0 (SPSS Inc., Chicago, IL, United States). The maximum total score of the WPI and SSS is 19 and 12, respectively. SSS is the sum of the three-symptom severity (fatigue, wakefulness, and cognitive symptoms) plus the sum of the number of the following symptoms that have occurred within the previous six months: headache, lower abdominal pain or cramps, and depression (0–3). A patient was considered to meet the modified ACR 2010 fibromyalgia diagnostic criteria if the following three conditions were met [26]:

1. WPI of 7 or more, and SSS ≥ 5, or WPI between 3–6, and SSS ≥ 9.
2. Symptoms were present at a similar level for at least three months.
3. The patient does not have a disorder that would explain the pain.

All variables (including case and control groups) were described using frequency distributions. Binomial logistic regression was performed to ascertain the effects of gender, marital status, residence, employment status, health insurance, financial status, currently diagnosed with major depressive disorder, general anxiety disorder, irritable bowel syndrome, and restless legs syndrome on the likelihood that participants have fibromyalgia. A *P*-value ≤ 0.05 was considered statistically significant.

#### Ethical consideration

The Research Ethics Committee in the Syrian Private University, and the ethical committees in the concerned hospitals approved the study protocol. Informed consent was obtained from each participant prior to participation.

## Results

### Participants’ characteristics

Out of the 2072 participants who took the in-person survey and 1011 who completed the online survey, 102 and 15 respondents, respectively, were excluded for not meeting the inclusion criteria, resulting in a cohort of 2966 participants (response rate 96.2%). Of these, 1315 (44.3%) were males and 1651 (55.7%) were females. Regarding the age groups, most participants [1720 (58%)] were 18—29 years old, 1035 (34.9%) were 30—49 years old, and 211 (7.1%) were ≥ 50 years. The majority were singles 1544 (52.1%), and 1422 (48%) were in a relationship. Unemployed individuals represented half of our sample [1502 (50.6%)]. The majority [2548 (85.9%)] did not have health insurance, and 2412 (81.3%) lived in a city. Regarding financial status, 1514 (51.1%), and 1452 (49%) were below average and had average financial status, respectively. When asked about previous medical history, 29 (0.9%) said that they were diagnosed with fibromyalgia, and 30 (1%), 163 (5.5%), 268 (15.3%), and 64 (3.7%) said they have systemic lupus erythematosus (SLE), rheumatoid arthritis (RA), irritable bowel syndrome (IBS) and restless leg syndrome (RLS), respectively. (Table.1).

**Table 1:** Participants’ characteristics: (n = 2966)

Variables	Total (2966) N(%)	FM (350) N(%)	Non-FM (2616) N(%)
*Age (Years)*			
18–29	1720 (58)	250 (71.4)	1470 (56.2)
30–49	1035 (34.9)	84 (24)	951 (36.4)
≥ 50	211 (7.1)	16 (4.6)	195 (7.5)
*Gender*			
Male	1315 (44.3)	111 (31.7)	1204 (46)
Female	1651 (55.7)	239 (68.3)	1412 (54)
*Marital status*			
Single	1544 (52.1)	199 (56.9)	1345 (51.4)
In a relationship	1422 (48)	151 (43.1)	1271 (48.6)
*employment status*			
Unemployed	1502 (50.6)	237 (67.7)	1265 (48.3)
Employed	1768 (59.6)	146 (41.7)	1622 (62)
*Do you have health insurance?*			
No	2548 (85.9)	266 (76)	2282 (87.2)
Yes	418 (14.1)	84 (24)	334 (12.8)
*Current residence*			
City	2412 (81.3)	288 (82.3)	2124 (81.2)
Rural	554 (18.7)	62 (17.7)	492 (18.8)
*Financial status*			
Below average	1514 (51.1)	127(36.2)	1387 (53)
Average and above	1452 (49)	223 (63.7)	1229 (47)
*What is your highest education level?*			
Elementary	149 (5)	14 (4)	135 (5.2)
Post-university	2817 (94.9)	336 (95.9)	2481 (94.8)
*Currently diagnosed with Fibromyalgia*			
No	2937(99.1)	321(91.7)	2616(100)
Yes	29(0.9)	29(8.2)	0(0)
*Currently diagnosed with systemic lupus erythematous*			
No	2936 (99)	346 (98.9)	2590 (99)
Yes	30 (1)	4 (1.1)	26 (1)
*Currently diagnosed with rheumatoid arthritis*			
No	2803 (94.5)	308 (88)	2495 (95.4)
Yes	163 (5.5)	42 (12)	121 (4.6)
*Currently diagnosed with other immune syndromes*			
No	2802 (94.5)	309 (88.3)	2493 (95.3)
Yes	163 (5.5)	41 (11.7)	123 (4.7)
*Currently diagnosed with major depressive disorder*			
No	2741 (92.4)	281 (80.2)	2460 (94)
Yes	225 (7.5)	69 (19.7)	156 (6)
*Currently diagnosed with general anxiety disorder*			
No	2677 (90.2)	281 (80.2)	2396 (91.5)
Yes	289 (9.7)	69 (19.8)	220 (8.5)
*Currently diagnosed with irritable bowel syndrome*			
No	2535 (85.4)	260 (74.2)	2276 (87)
Yes	431 (14.5)	90 (25.8)	340 (12.9)
*Currently diagnosed with restless legs syndrome*			
No	2846 (95.9)	278 (79.4)	2568 (98.1)
Yes	120 (4.1)	72 (20.5)	48 (1.8)

### Prevalence of fibromyalgia

In total, 350 (11.8%) participants were found to meet the modified ACR 2010 criteria. Of these, 239 (68.3%) were females. The majority [250 (71.4%)] were 18—29 years old, single [199 (56.9%)], unemployed [237 (67.7%)], and did not have health insurance [266 (76%)]. Only 62 (17.7%) lived in rural areas. Regarding financial status, 127 (36.2%), and 223 (63.7%) were below average and had average financial status, respectively. When asked about previous medical history, only 29(8.2%) said that they were diagnosed with fibromyalgia, and 4 (1.1%), 41 (11.7%), 90 (25.8%), and 72 (20.5%) said they have systemic lupus erythematosus (SLE), rheumatoid arthritis (RA), irritable bowel syndrome (IBS) and restless leg syndrome (RLS), respectively. On the other hand, an equal number of participants had the diagnosis of major depressive disorder [69 (19.8%)], and general anxiety disorder [69 (19.8%)], respectively (Table 1).

When asked about the number of sites that involved any pain problem in the last week, [45 (12.9%)] stated that they had problems in 3 to 6 sites, and [305 (87.1%)] had problems in 7 or more sites.

### The associations of the study variables among cases and controls

The logistic regression model was statistically significant, x^2^(11) = 124.929, *P*-value < 0.001. The model explained 10.9% (Nagelkerke R2) of the variance in fibromyalgia and correctly classified 80.1% of the cases. Of the 10 variables, only 6 were statistically significant: gender, employment status, health insurance, currently diagnosed with major depressive disorder, irritable bowel syndrome, and restless legs syndrome. females had 1.31 higher odds to have fibromyalgia than males. Employment was associated with a reduction in the likelihood of having fibromyalgia. Having health insurance was associated with 1.96 higher odds of having fibromyalgia. Regarding associated diseases, diagnosis of major depressive disorder (OR = 2.62), Irritable bowel syndrome (OR = 1.8), and Restless legs syndrome (OR = 1.72) were associated with a higher likelihood of fibromyalgia. (Table 2).

**Table 2:** Logistic regression: predicting the likelihood of fibromyalgia based on study characteristics

Variables	Odds ratio (95% CI)	*P*-value
*Gender*		
Female	1.31 (1–1.71)	0.048*
Male	Reference	
*Marital status*		
Married, in a relationship,	0.99 (0.77–1.28)	0.983
Single	Reference	
*Residence*		
Rural	0.91 (0.66–1.25)	0.572
City	Reference	
*Employment status*		
Employed	0.76 (0.58–0.99)	0.045*
Unemployed	Reference	
*Do you have health insurance?*		
Yes	1.96 (1.43–2.70)	< 0.001*
No	Reference	
*Financial status*		
Good (Average, Good, Excellent)	1.15 (0.88–1.52)	0.288
Bad (Bad, Below average)	Reference	
*Currently diagnosed with major depressive disorder*		
Yes	2.62 (1.72–3.98)	< 0.001*
No	Reference	
*Currently diagnosed with general anxiety disorder*		
Yes	1.34 (0.89–2)	0.154
No	Reference	
*Currently diagnosed with Irritable bowel syndrome*		
Yes	1.8 (1.32–2.45)	< 0.001*
No	Reference	
*Currently diagnosed with Restless legs syndrome*		
Yes	1.72 (1.00–2.98)	0.050*
No	Reference	

*P-value ≤ 0.05 was considered statistically significant

## Discussion

Our study is the first, to our knowledge, to address FM prevalence and the basic characteristics of FM patients and to identify the risk factors in the general population in Damascus, Syria. Our results found that 350 (11.8%) have satisfied the ACR 2010 modified criteria of FM diagnosis, which represents one of the highest prevalence rates of FM in a general population. When considering that many studies showed a psychological predisposition to FM [2, 27], our finding could be explained by the huge psychological distress experienced by the Syrian population. Syria has been at war for more than a decade now. This led to huge consequences with more than 6 million people being internally displaced to safer areas like Damascus city. Moreover, the economy experienced great damage resulting in 50% of working-age people becoming unemployed, and more than 90% of people falling under the poverty line. More than 13.4 million Syrian people are in need, 1.1 million of which are in Damascus [28, 29]. Finally, the COVID-19 pandemic has had a devastating effect on Syrian society. The impact of the war on the prevalence of FM was evident in a study conducted on Syrian female refugees in Jordan that revealed a prevalence of severe FM at about 30% [30].

Undiagnosed FM may result in inadequate treatment and relief of key symptoms like pain, fatigue, and unrefreshed sleep. This will mislead medical decisions to change or escalate the dosage of treatments of the underlying disease rather than addressing FM. In our study, only 29 (8.2%) of the 350 participants who satisfied the ACR 2010 modified criteria were diagnosed by a medical professional. A similar observation was made in a study conducted in Lebanon, as all FM cases were previously misdiagnosed and did not receive a diagnosis of FM [13]. The diagnosis of FM requires the efforts of patients to seek medical care and clinicians who are familiar with the disease and are willing to make the diagnosis, as FM diagnosis is confounded by drug-seeking behavior and malingering [31].

Our study shows that females have greater odds to have FM (OR = 1.31), which goes in line with the literature [9, 11–13]. Having health insurance is shown to be associated with a higher likelihood of FM in our population, probably because health insurance is not common in Syria, as only 14.1% of our sample have it. In addition, this insurance is offered to the workers in the public sector that have low wages due to economical inflation. One study showed that occupational stress is considered a risk factor for FM [32], while our results showed that working participants have lower odds of having FM, it was contrary to the results of a Lebanese study that indicated work is a risk factor [13]. This may be due to the bad economic status in Syria, which made it difficult to secure the basics of living, thus making unemployment very stressful. Surprisingly, there was no significant association between income and FM., and this was inconsistent with what was reported in studies conducted in Europe and Lebanon [11, 13]. This is probably because the question about the financial status has been left subjective to the assessment of the participants, which may vary between the participants according to their opinions, making the results unreliable.

In our study, 225 and 289 of our participants stated that they are diagnosed with major depressive disorder and/or generalized anxiety disorder. However, a recent national study conducted on the general population in Syria found that mental health disorders have reached high rates, with rates of depression and anxiety reaching 83.4% and 69.6%, respectively [33]. This may represent a very concerning proportion, because of the fact that there is growing literature addressing a possible link between FM and many psychological disorders [5, 12, 34]. Our study shows that major depressive disorder (OR = 2.62) is associated with a higher likelihood of FM. While there was no statistically significant association with generalized anxiety disorder. other studies showed association with both of the aforementioned disorders as indicated in a US study with depression (OR = 2.85) and anxiety (OR = 3.47) found to be associated with a higher likelihood of FM females with FM [35]. Gastrointestinal comorbidities are also common in FM patients. We found that IBS patients had a higher likelihood (OR = 1.8) of having FM. A similar finding was demonstrated in a study in Norway conducted on IBS patients that found that FM was associated with higher odds (OR = 3.6) for IBS [36].

FM is associated with a high burden of illness on patients and the healthcare system, which causes significant disability [19]. We encourage a general awareness program targeting healthcare professionals and the public to help early detection of the disease and ensure a smoother and safer treatment journey specifically directed towards the symptoms of the disease and improvement of patients’ quality of life. In addition, Long-term prospective studies should be conducted to address the patterns of treatment, financial burden, and productivity loss among patients.

## Limitation

As many of our participants were young in age, we think that The demographic distribution of our sample is relatively representative of Syrian society according to data from the Central Bureau of Statistics (CBS), Damascus, Syria. that documented in its last latest report that ~ 40% of the Syrian population were below 24 years old, 25.5% were 25–44 years old, 17.6% were 45–65 years old, and 5.3% were above 65 years old [24]. We tried to minimize the selection bias by distributing the questionnaire online and in hard copies, and getting a representative sample of people with low economic status, as the online questionnaire may be filled mostly by young age groups with a good economic level. In addition, surveying participants from the hospital might lead to a higher percentage of patients with FM due to the fact that their sickness might put a huge psychological stress burden on the patients and their families. Recall bias is present due to self-reporting. The economic status question was left subjective because the Syrian currency is not stable and is changing dramatically on a daily basis. Some aspects that might have accounted for differences in the odds of the disease were not addressed, such as the use of oral contraceptive pills, family history, and body mass index.

## Conclusion

FM appears to be a very common disease in Damascus, Syria. The lack of knowledge regarding the disease has resulted in many patients suffering due to the lack of recognition of the disease by patients and physicians alike.

## Supplementary Information

**Additional file 1**. Stage one database. Include the participants who met the inclusion criteria for stage one.

**Additional file 2**. Stage two database. Include the cases and controls that were analyzed in stage two.

## Data Availability

The datasets used and/or analyzed during the current study are available as supplementary material in the appendix. (Additional file 1) for stage one, and (Additional file 2) for stage two.
